# Supplementary material for: Prognostic Value and Function of KLF5 in Papillary Thyroid Cancer
Source: Cancers (Basel). 2021 Jan 7;13(2):185. doi: 10.3390/cancers13020185 (PMC7825749; doi:10.3390/cancers13020185)

cancers

MDPI

Supplementary material

Prognostic Value and Function of KLF5 in Papillary Thyroid Cancer

Poyil Pratheeshkumar [1,†], Abdul K. Siraj [1,†], Sasidharan Padmaja Divya [1], Sandeep Kumar Parvathareddy [1], Sarah Siraj [1], Roxanne Diaz [1], Rafia Begum [1], Saif S. Al-Sobhi [2], Fouad Al-Dayel [3] and Khawla S. Al-Kuraya [1,*]

1 Human Cancer Genomic Research, Research Center, King Faisal Specialist Hospital and Research Center, P.O. Box 3354, Riyadh 11211, Saudi Arabia; ppoyil@kfshrc.edu.sa (P.P.); asiraj@kfshrc.edu.sa (A.K.S.); pdivya@kfshrc.edu.sa (S.P.D.); psandeepkumar@kfshrc.edu.sa (S.K.P.); sarah-siraj@kfshrc.edu.sa (S.S.); rmelosantos87@kfshrc.edu.sa (R.D.); brafia@kfshrc.edu.sa (R.B.)

2 Department of Surgery, King Faisal Specialist Hospital and Research Center, P.O. Box 3354, Riyadh 11211, Saudi Arabia; sobhi@kfshrc.edu.sa

3 Department of Pathology, King Faisal Specialist Hospital and Research Centre, P.O. Box 3354, Riyadh 11211, Saudi Arabia; dayelf@kfshrc.edu.sa

* Correspondence: kkuraya@kfshrc.edu.sa; Tel.: +(966)-1-205-5167

† These authors contributed equally to this work.

Citation: Pratheeshkumar, P.; Siraj, A.K.; Divya, S.P.; Parvathareddy, S.K.; Siraj, S.; Diaz, R.; Begum, R.; Al-Sobhi, S.S.; Al-Dayel, F.; Al-Kuraya, K.S. Prognostic Value and Function of KLF5 in Papillary Thyroid Cancer. *Cancers* **2021**, *13*, 185. https://doi.org/10.3390/cancers13020185

Received: 1 December 2020
Accepted: 1 January 2021
Published: 7 January 2021

Publisher's Note: MDPI stays neutral with regard to jurisdictional claims in published maps and institutional affiliations.

Copyright: © 2021 by the authors. Submitted for possible open access publication under the terms and conditions of the Creative Commons Attribution (CC BY) license (http://creativecommons.org/licenses/by/4.0/).

Supplementary materials

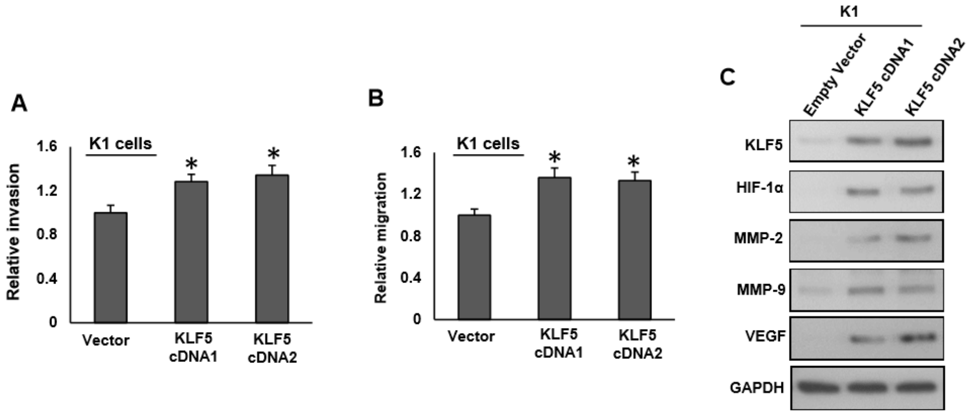

Figure 1. Forced expression of KLF5 increased invasive and migratory potential in PTC cells. (A) Forced expression of KLF5 increased cell invasion. K1 cells were. transfected with either empty vector or KLF5 cDNA for 48 hours, cells were seeded into the upper compartment of invasion chambers. The bottom chambers were filled with RPMI media. After 24 h incubation, invaded cells were fixed, stained and quantified. (B) Forced expression of KLF5 increased cell migration. K1 cells were transfected with either empty vector or KLF5 cDNA for 48 hours, cells were seeded into the upper compartment of migration chambers. The bottom chambers were filled with RPMI media. After 24 h incubation, migrated cells were fixed, stained and quantified. (C) Forced expression of KLF5 increased MMP-2, MMP-9 and VEGF expression. K1 cells were transfected with either empty vector or KLF5 cDNA for 48 hours. Proteins were isolated and immunoblotted with antibodies against KLF5, HIF-1α, MMP-2, MMP-9, VEGF and GAPDH. Data presented in the bar graphs are the mean ± SD of two independent experiments. *Indicates a statistically significant difference compared to control with $p < 0.05$.

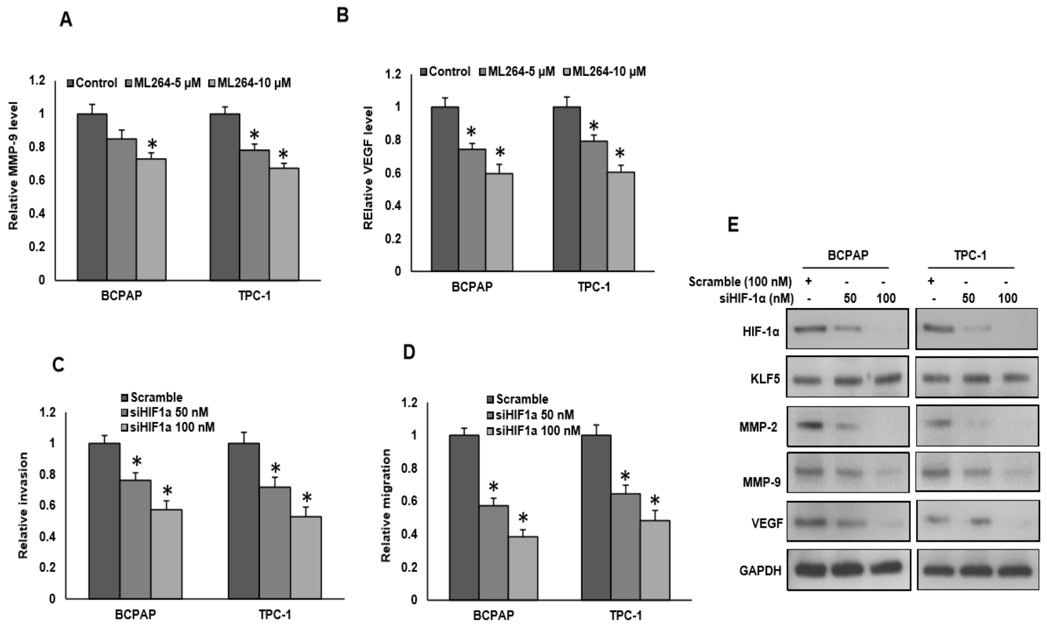

Figure 2. Inhibition of KLF5 and HIF-1α decreased the invasive and migratory potential in PTC cells. (A) Inhibition of KLF5 decreased MMP9 secretion in PTC cells. PTC cells.were treated with indicated doses of ML264 for 48 hours, and secreted MMP9 level in the media was estimated by MMP9 ELISA kit (R&D, Minneapolis, MN) according to the manufacturers' recommendations. (B) Inhibition of KLF5 decreased VEGF secretion in PTC cells. PTC cells were treated with indicatedose of ML264 for 48 hours, and secreted VEGF level in the media was estimated by VEGF ELISA kit (R&D, Minneapolis, MN) according to the manufacturers' recommendations. (C) Knockdown of HIF-1α reduced cell invasion. PTC cells were transfected with scrambled siRNA and HIF-1α siRNA (50 and 100 nM). After 48 hours, cells were seeded into the upper compartment of invasion chambers. The bottom chambers were filled with RPMI media. After 24 h incubation, invaded cells were fixed, stained and quantified. (D) Knockdown of HIF-1α reduced cell migration. PTC cells were transfected with scrambled siRNA and HIF-1α siRNA (50 and 100 nM). After 48 hours, cells were seeded into the upper compartment of migration chambers. The bottom chambers were filled with RPMI media. After 24 h incubation, migrated cells were fixed, stained and quantified. (E) Knockdown of HIF-1α downregulated the expressions of HIF-1α, MMP-2, MMP-9 and VEGF. PTC cells were transfected with scrambled siRNA and HIF-1α siRNA (50 and 100 nM). After 48 hours, cells were lysed and proteins were immunoblotted with antibodies against KLF5, HIF-1α, MMP-2, MMP-9, VEGF and GAPDH. Data presented in the bar graphs are the mean ± SD of two independent experiments. *Indicates a statistically significant difference compared to control with $p < 0.05$.

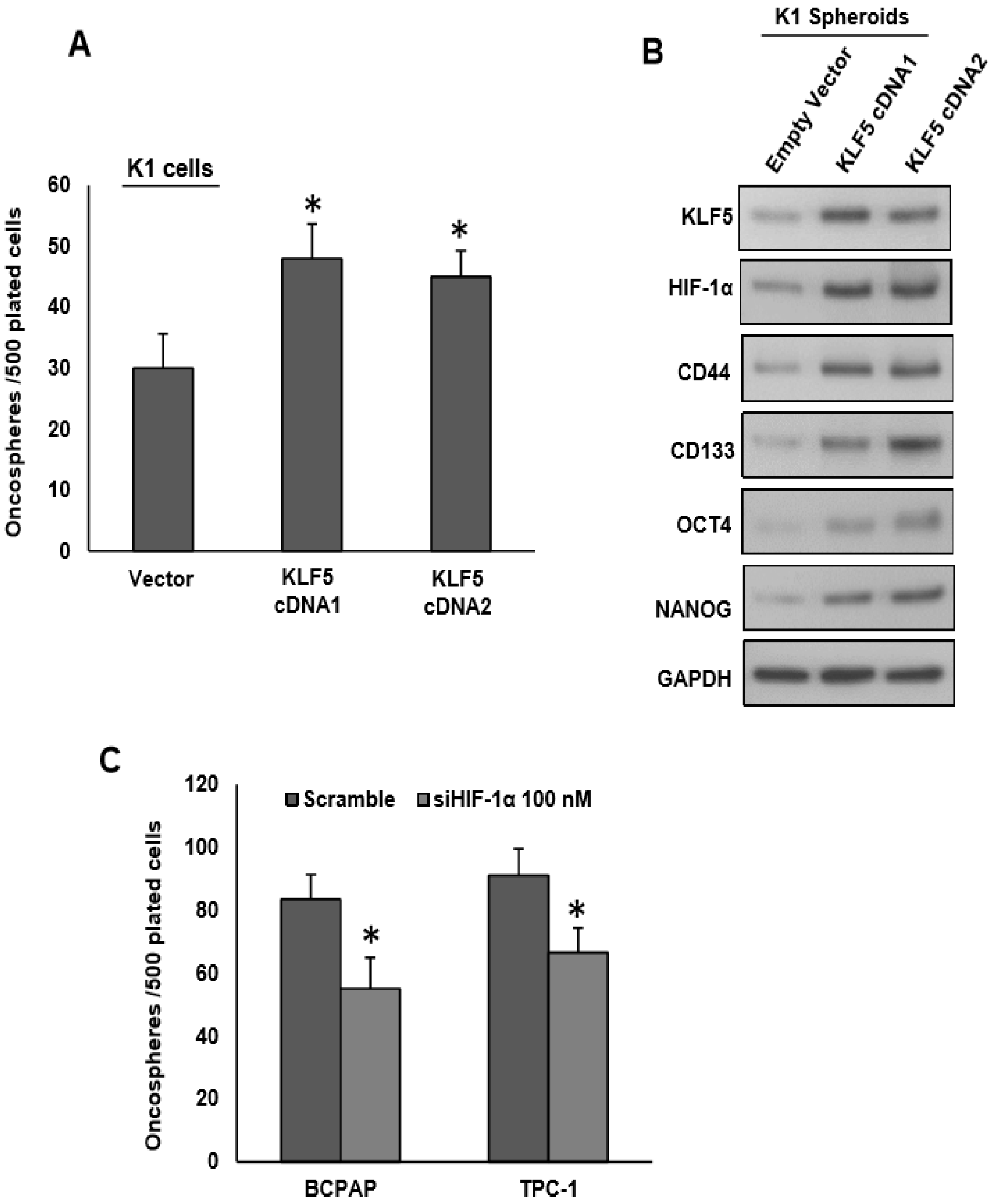

Figure 3. KLF5 and HIF-1α on stemness in PTC cells. (A) Forced expression of KLF5 increased self-renewal ability of spheroids. K1.cells were transfected with either empty vector or KLF5 cDNA and cells were subjected to sphere forming assay. Spheroids in the entire well were counted. (B) Forced expression of KLF5 increased stemness in PTC cells. K1 cells were transfected with either empty vector or KLF5 cDNA and grown in sphere medium. Proteins were isolated from spheroids and immunoblotted with antibodies against KLF5, HIF-1α, CD44, CD133, NANOG, OCT4 and GAPDH. (C) Silencing of HIF-1α decreased self-renewal ability of spheroids. PTC cells were transfected with scrambled siRNA and HIF-1α siRNA (100 nM) and cells were subjected to sphere forming assay. Spheroids in the entire well were counted. Data presented in the bar graphs are the mean ± SD of two independent experiments. *Indicates a statistically significant difference compared to control with $p < 0.05$.

Figure 2A.

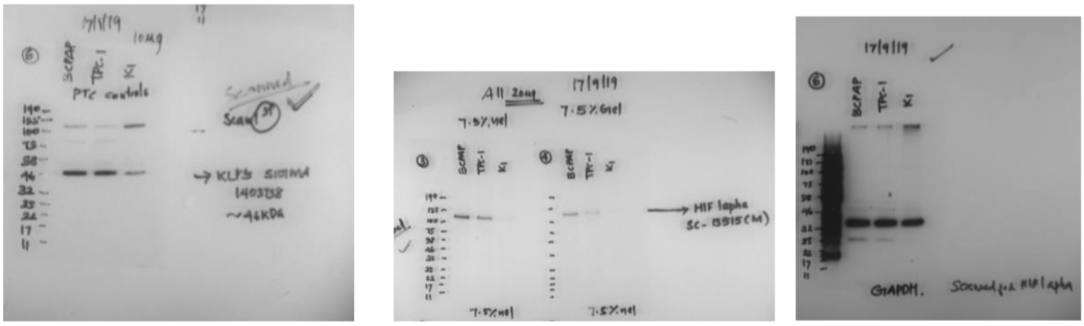

Figure 2B.

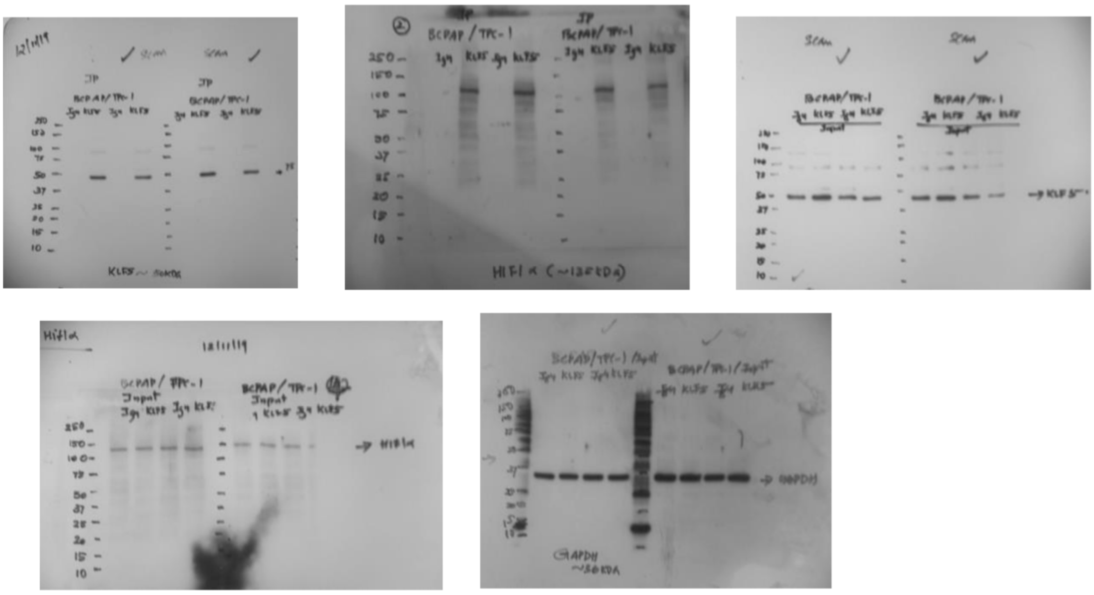

Figure 2C.

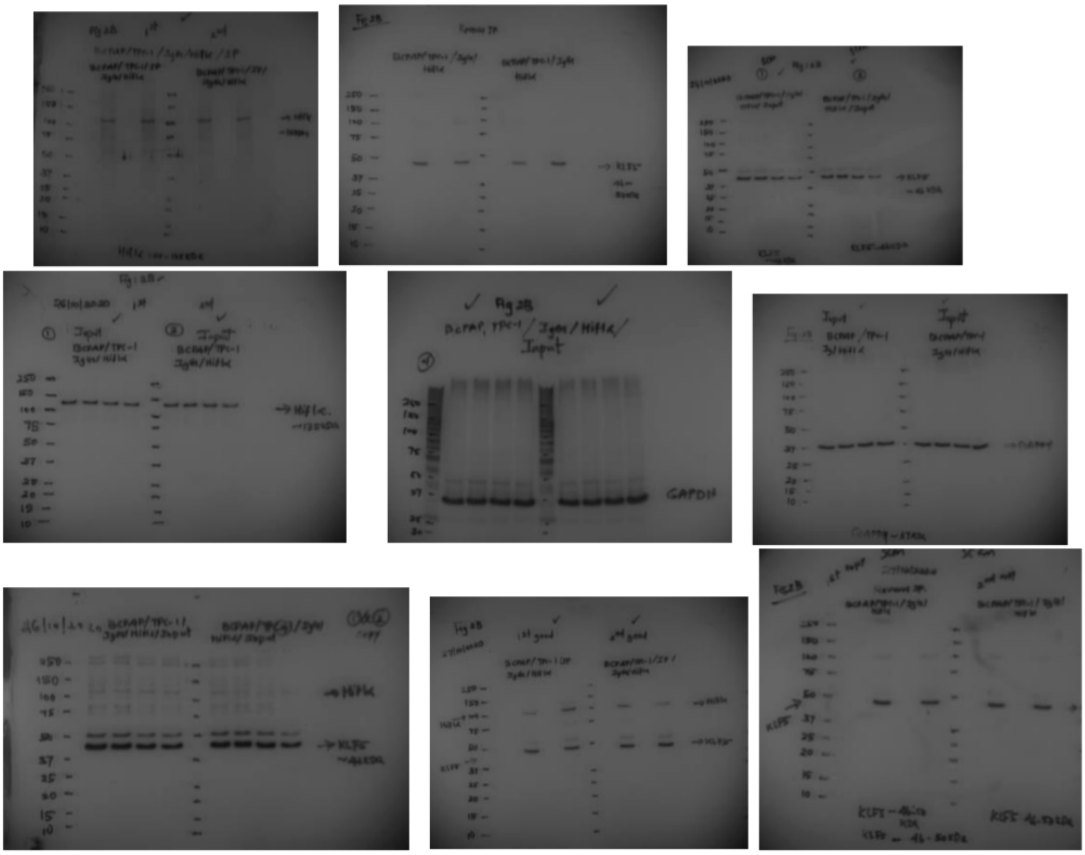

Figure 2D.

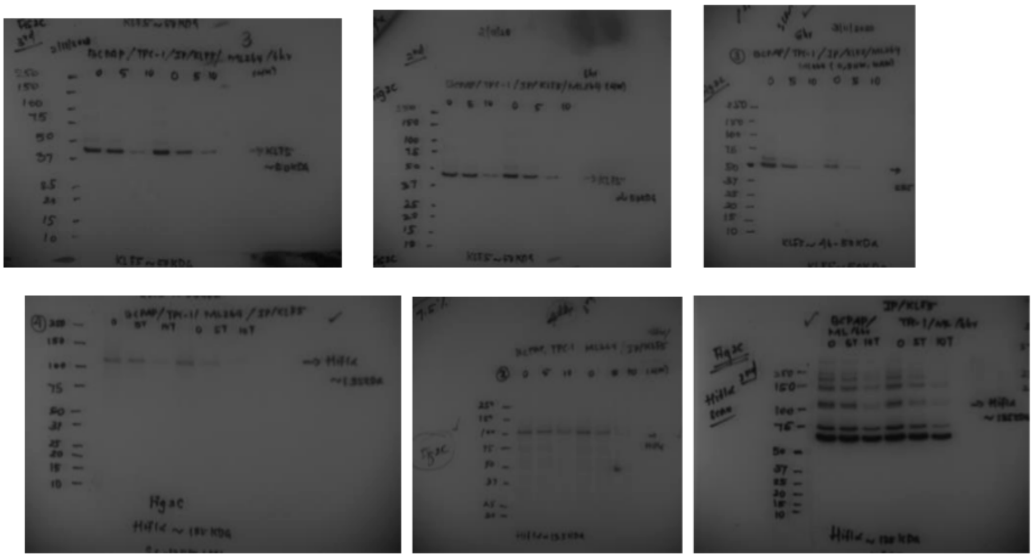

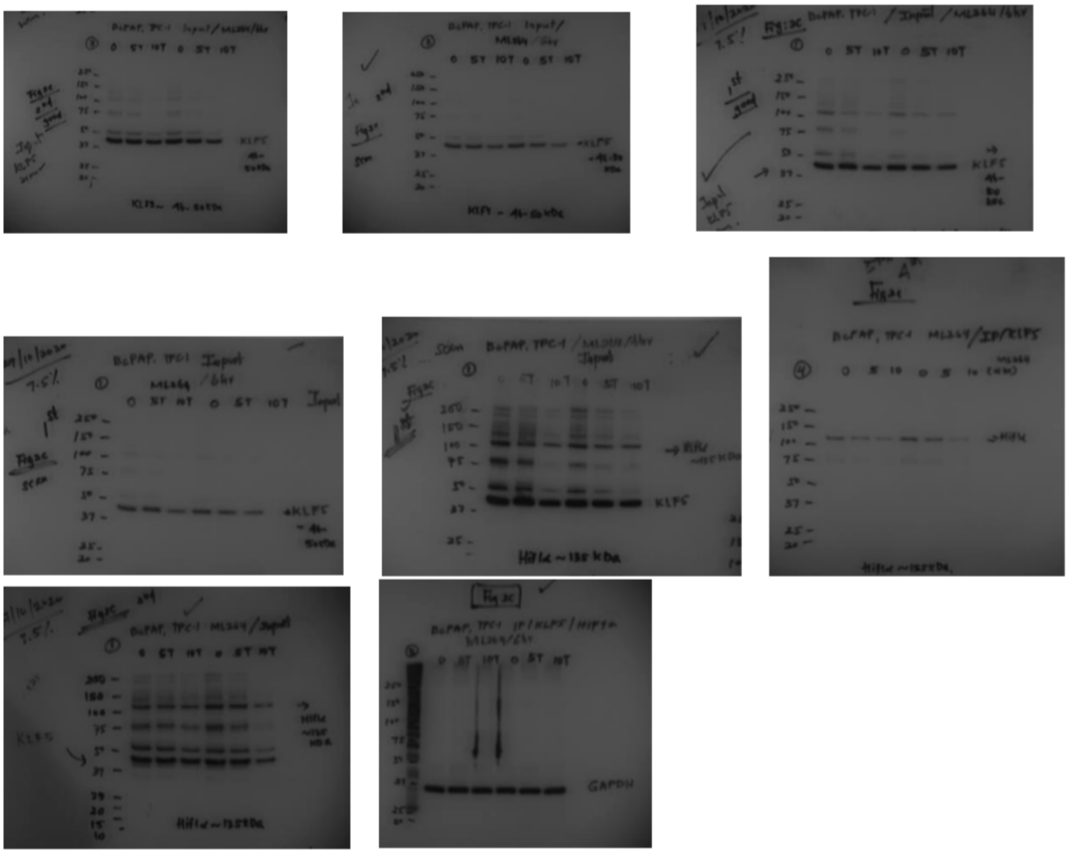

Figure 2E.

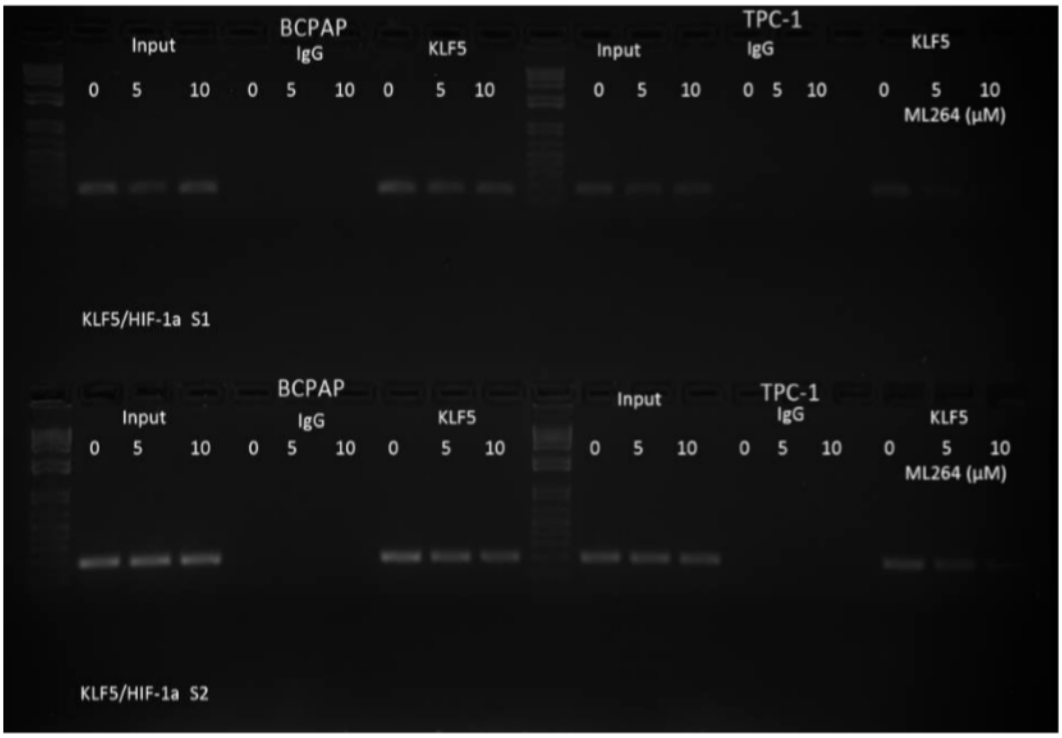
BCPAP
Input
IgG
KLF5
TPC-1
Input
IgG
KLF5
0 5 10 0 5 10 0 5 10 0 5 10 0 5 10 0 5 10
ML264 (µM)
KLF5/HIF-1a S1
BCPAP
Input
IgG
KLF5
Input
TPC-1
IgG
KLF5
0 5 10 0 5 10 0 5 10 0 5 10 0 5 10 0 5 10
ML264 (µM)
KLF5/HIF-1a S2

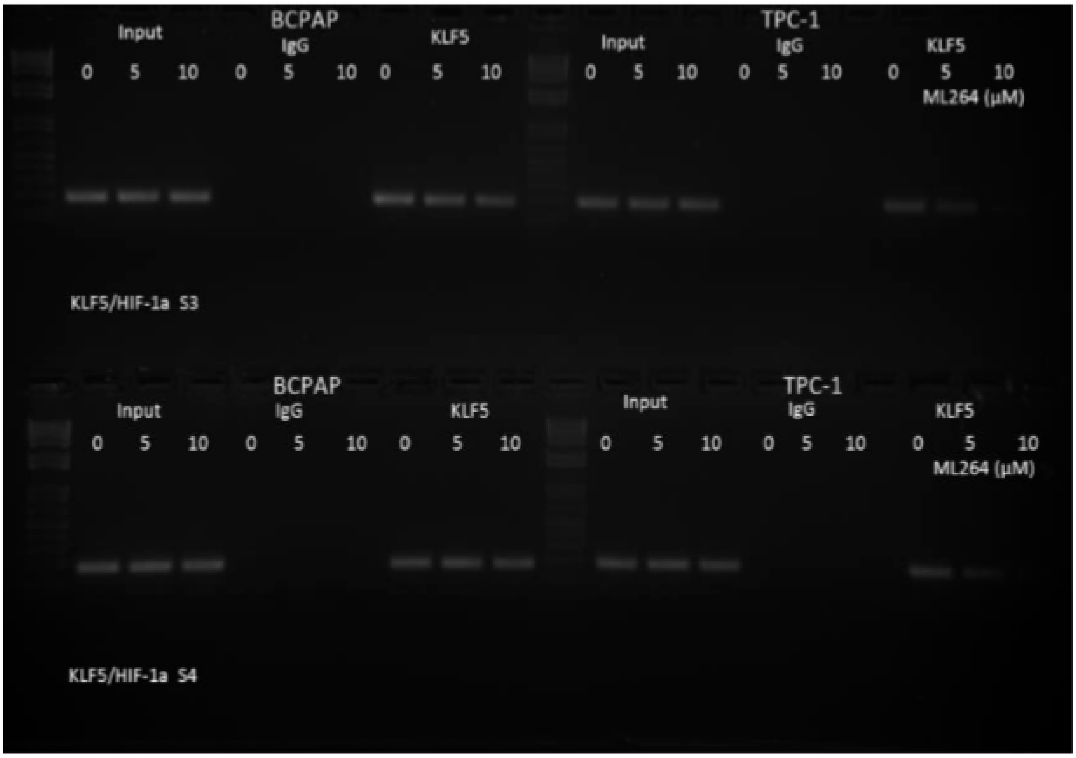
BCPAP
TPC-1
Input
IgG
KLF5
Input
IgG
KLF5
0 5 10 0 5 10 0 5 10 0 5 10 0 5 10 0 5 10
ML264 (µM)
KLF5/HIF-1a S3
BCPAP
TPC-1
Input
IgG
KLF5
Input
IgG
KLF5
0 5 10 0 5 10 0 5 10 0 5 10 0 5 10 0 5 10
ML264 (µM)
KLF5/HIF-1a S4

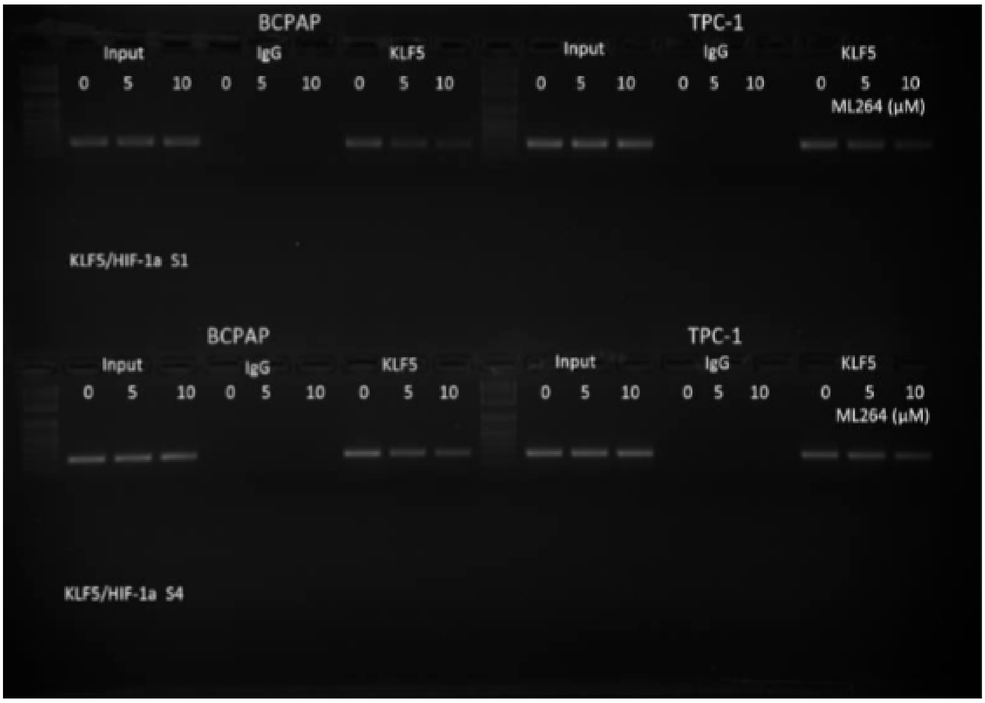
BCPAP
TPC-1
Input
IgG
KLF5
Input
IgG
KLF5
0 5 10 0 5 10 0 5 10 0 5 10 0 5 10 0 5 10
ML264 (µM)
KLF5/HIF-1a S1
BCPAP
TPC-1
Input
IgG
KLF5
Input
IgG
KLF5
0 5 10 0 5 10 0 5 10 0 5 10 0 5 10 0 5 10
ML264 (µM)
KLF5/HIF-1a S4

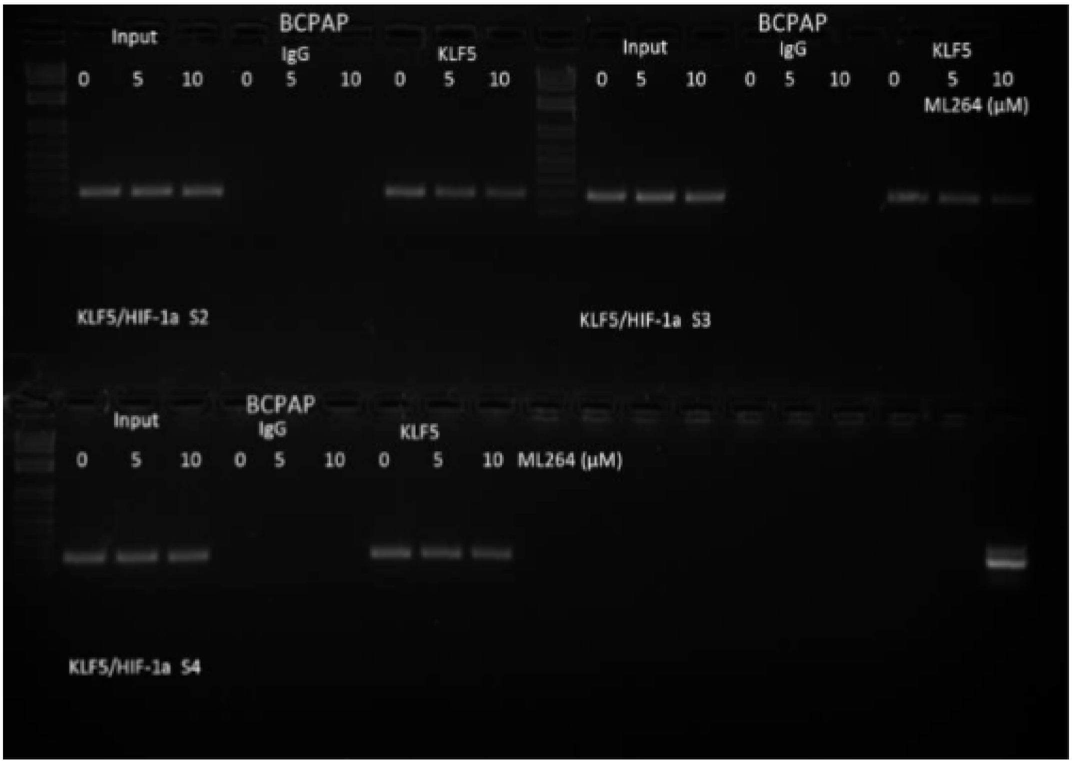
BCPAP
Input
IgG
KLF5
0 5 10 0 5 10 0 5 10
BCPAP
Input
IgG
KLF5
0 5 10 0 5 10 0 5 10
ML264 (µM)
KLF5/HIF-1a S2
KLF5/HIF-1a S3
BCPAP
Input
IgG
KLF5
0 5 10 0 5 10 0 5 10 ML264 (µM)
KLF5/HIF-1a S4

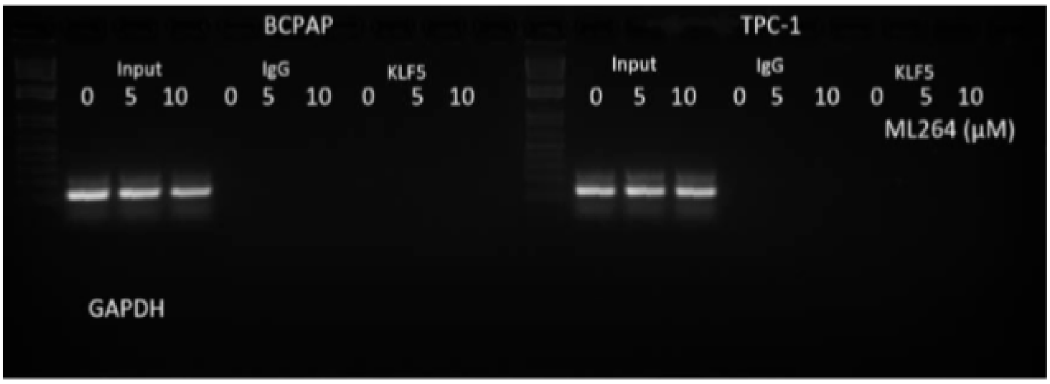
BCPAP
Input
IgG
KLF5
0 5 10 0 5 10 0 5 10
TPC-1
Input
IgG
KLF5
0 5 10 0 5 10 0 5 10
ML264 (µM)
GAPDH

Figure 2G.

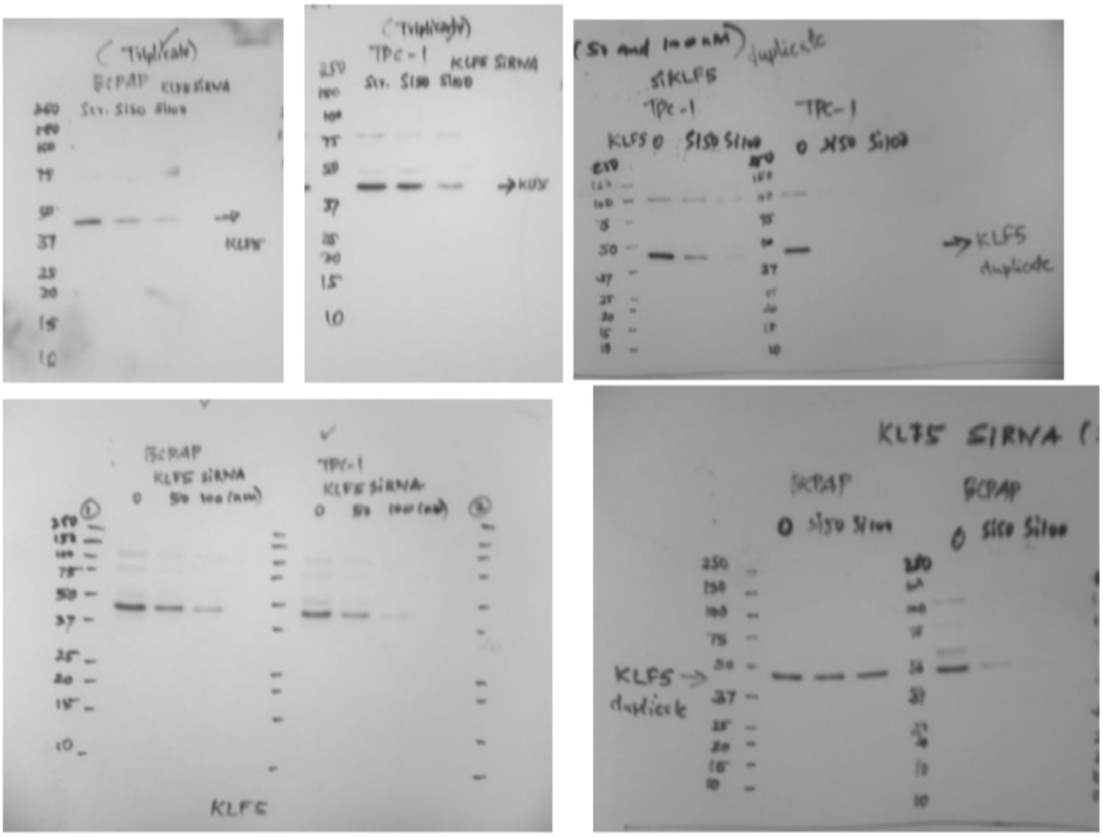

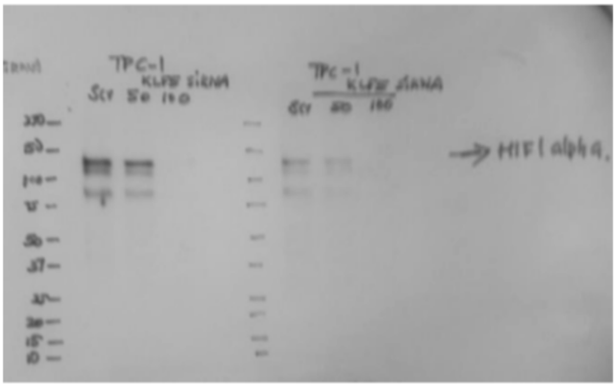
TPC-1
TPC-1
HIF1 alpha.

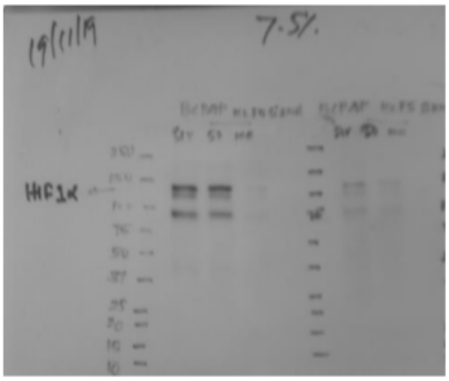
7.5%
HIF1α

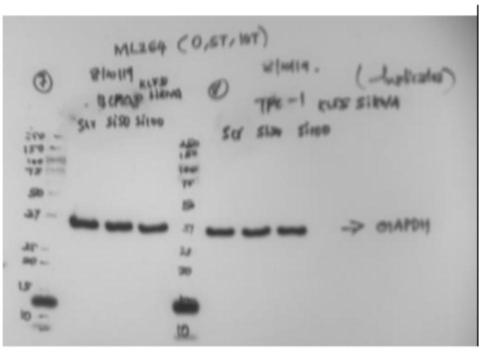
TPC-1 KLF5 SiRNA
GAPDH

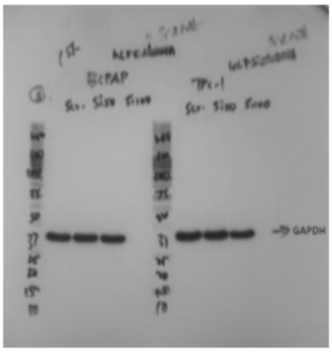
BCPAP
TPC-1
GAPDH

Figure 2H.

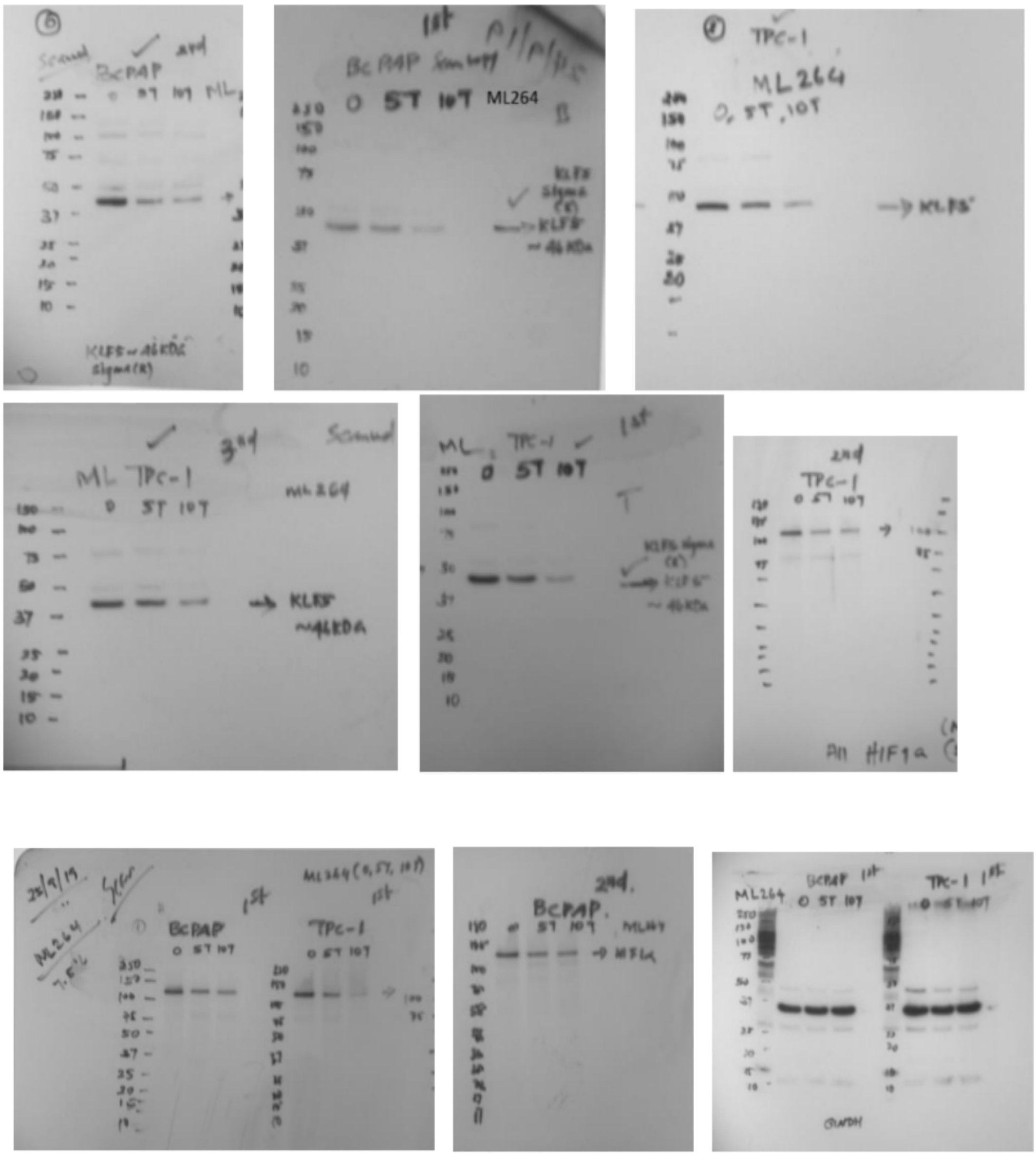

Figure 2I.

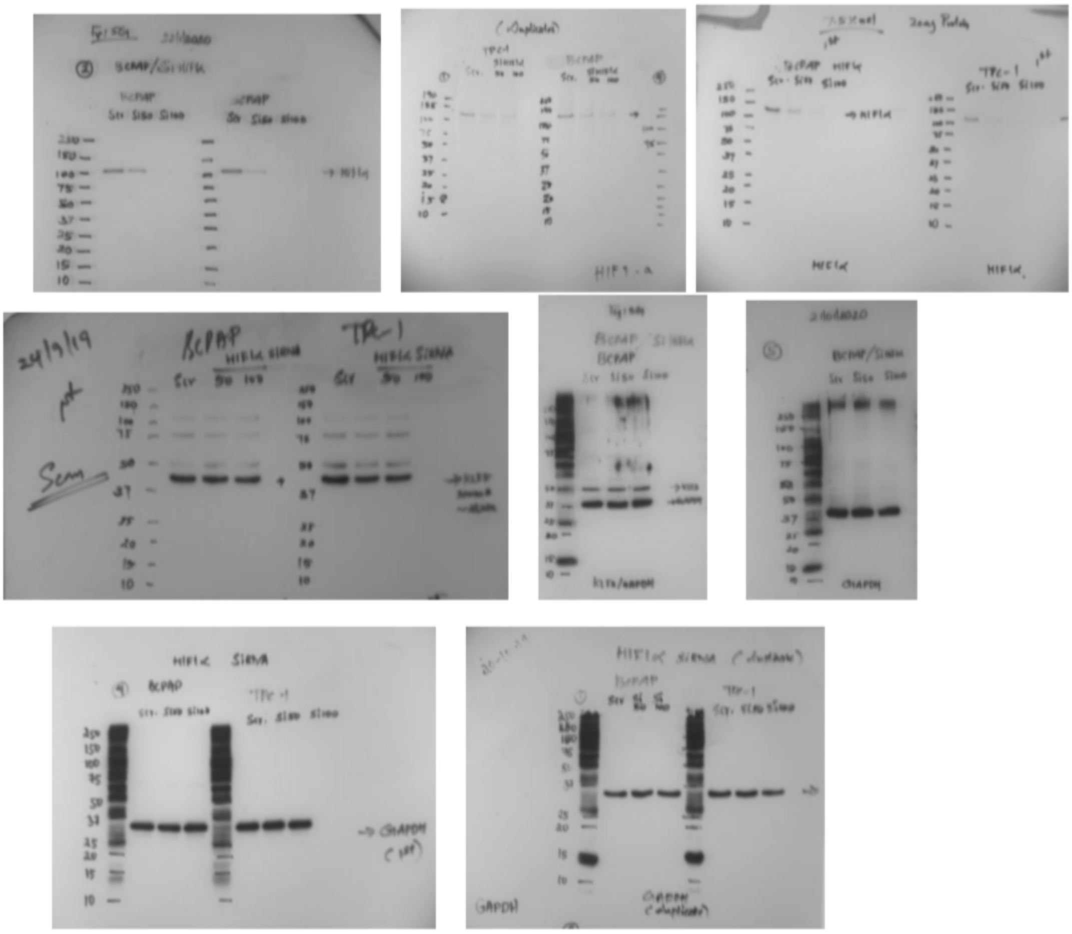

Figure 2J.

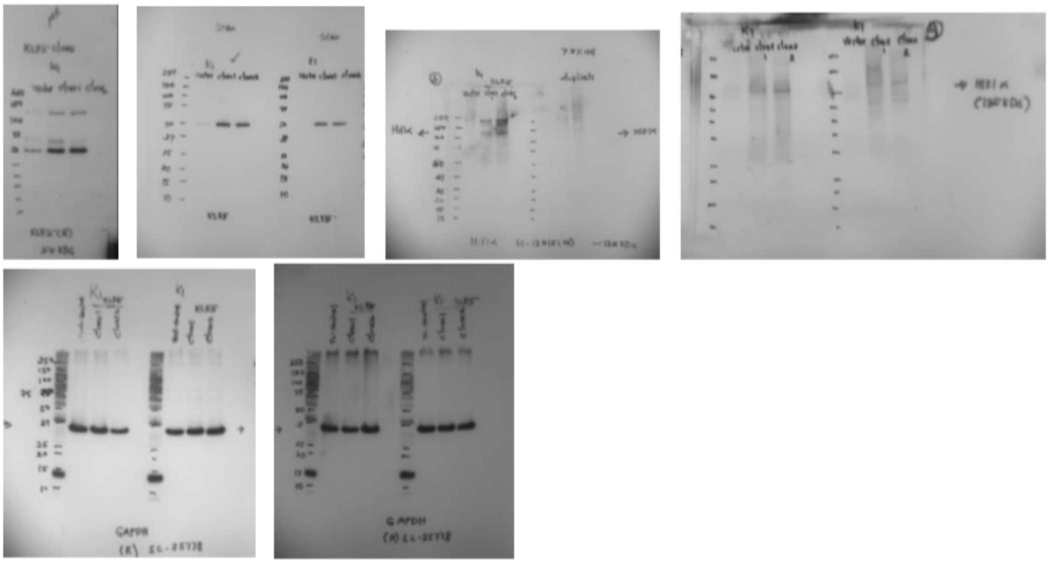

Figure 3F.

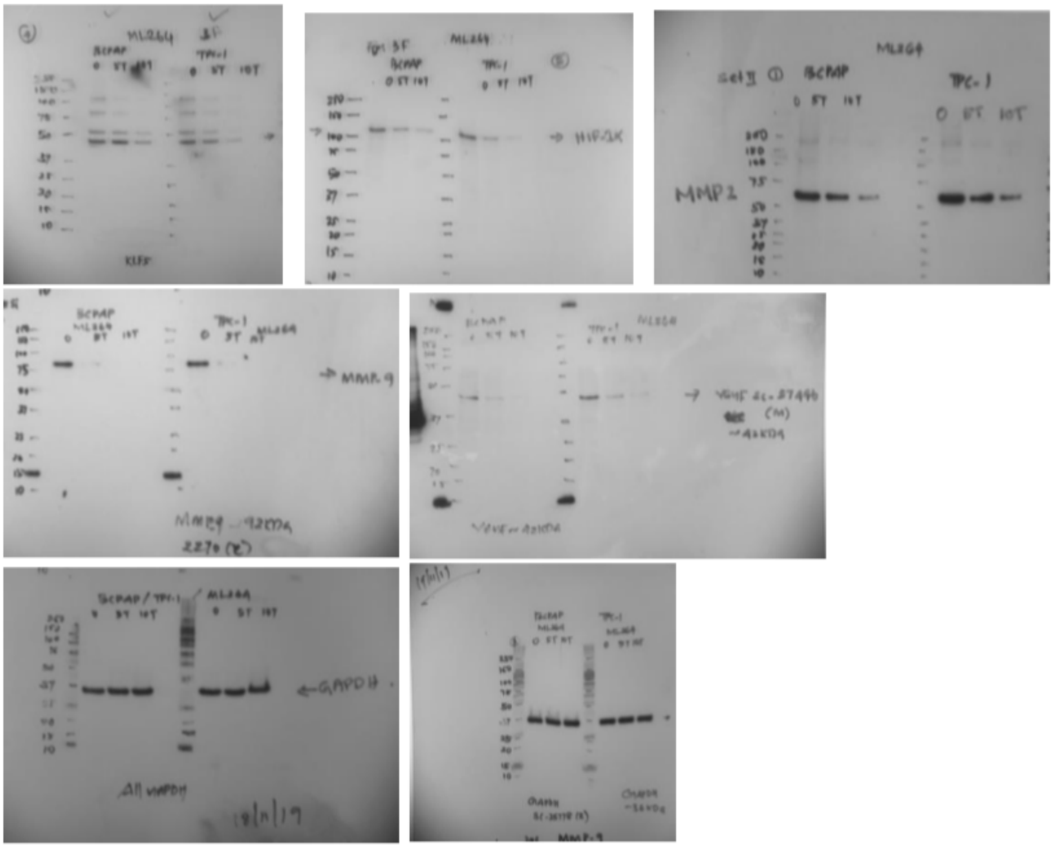

Figure 3G.

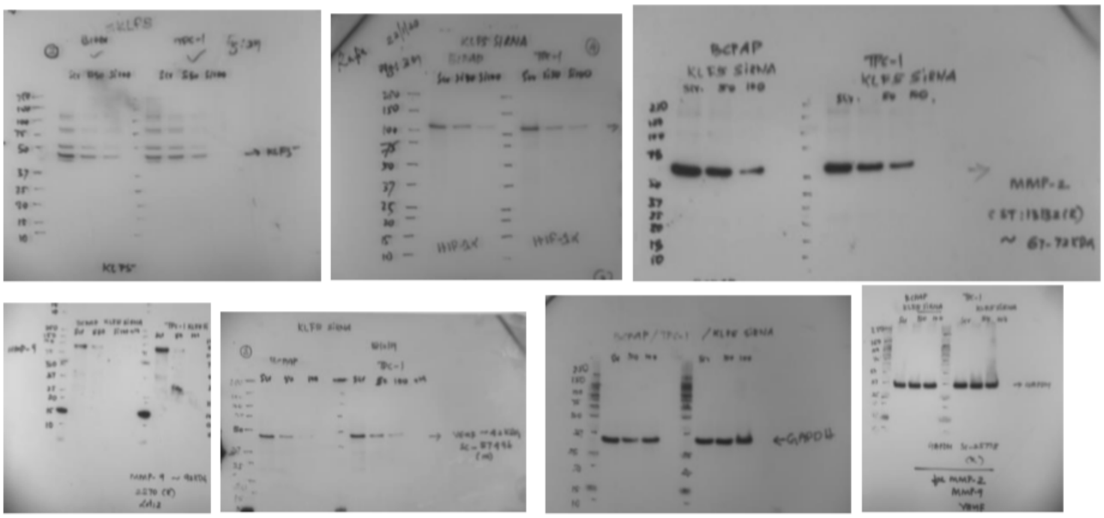

Figure 4F.

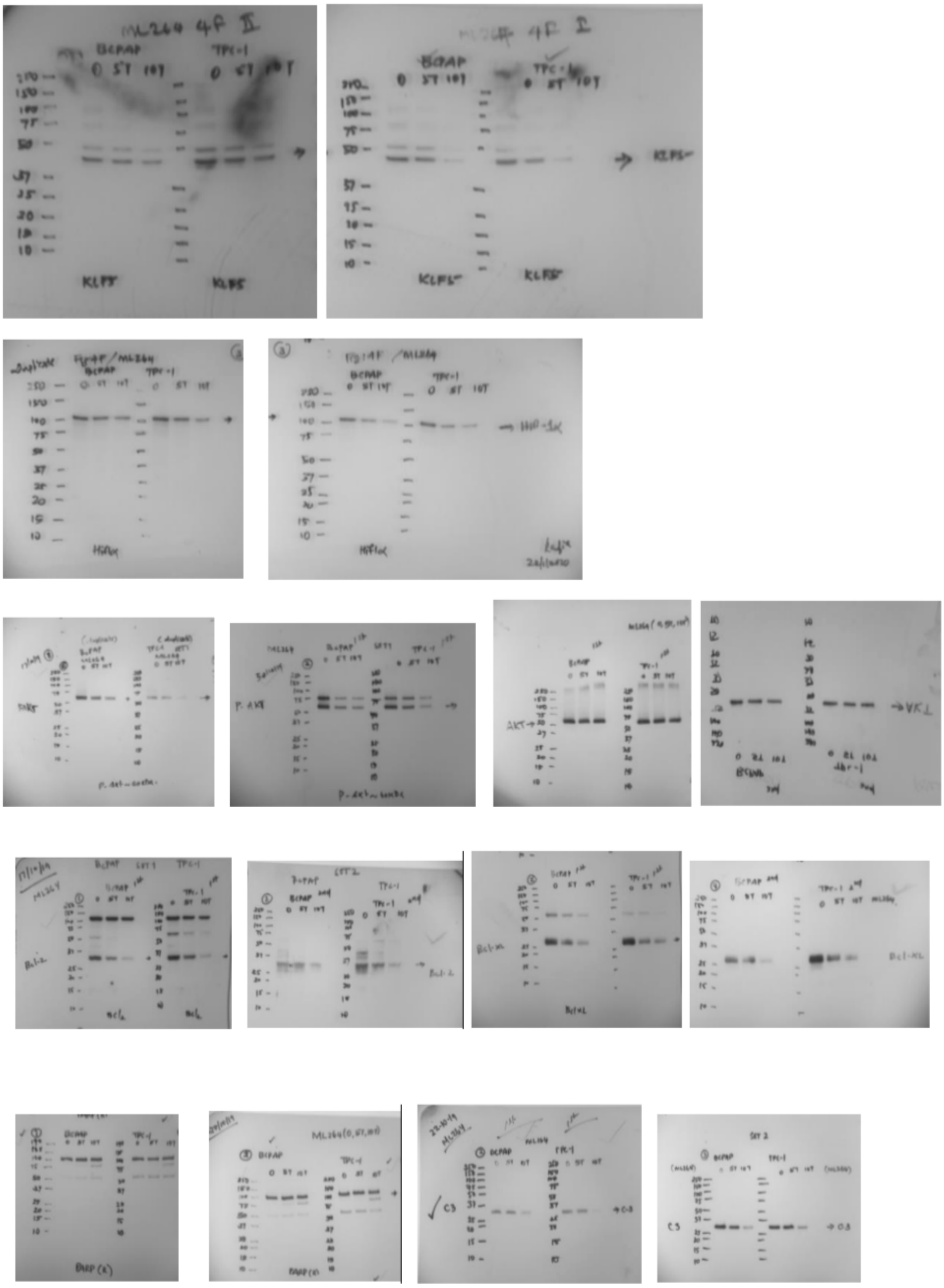

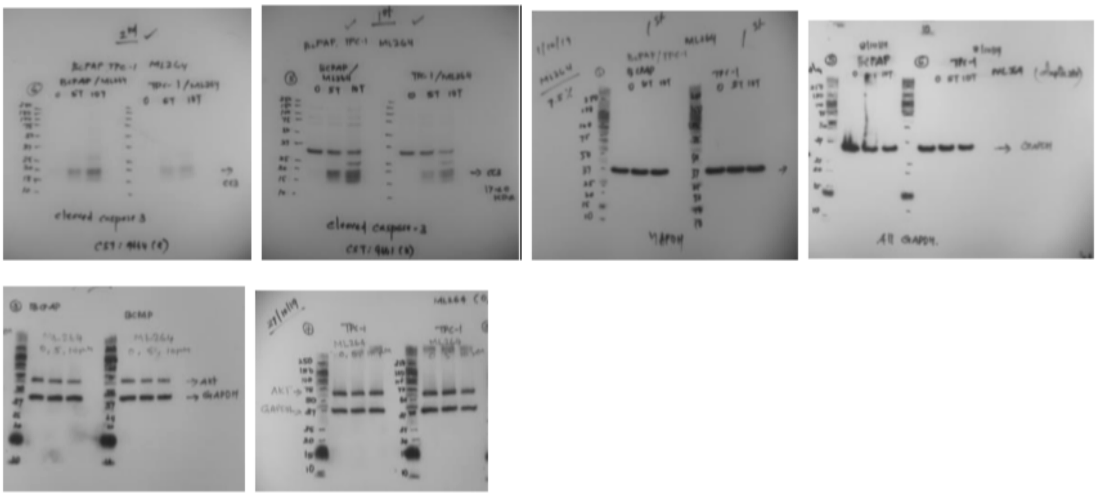

Figure 5A.

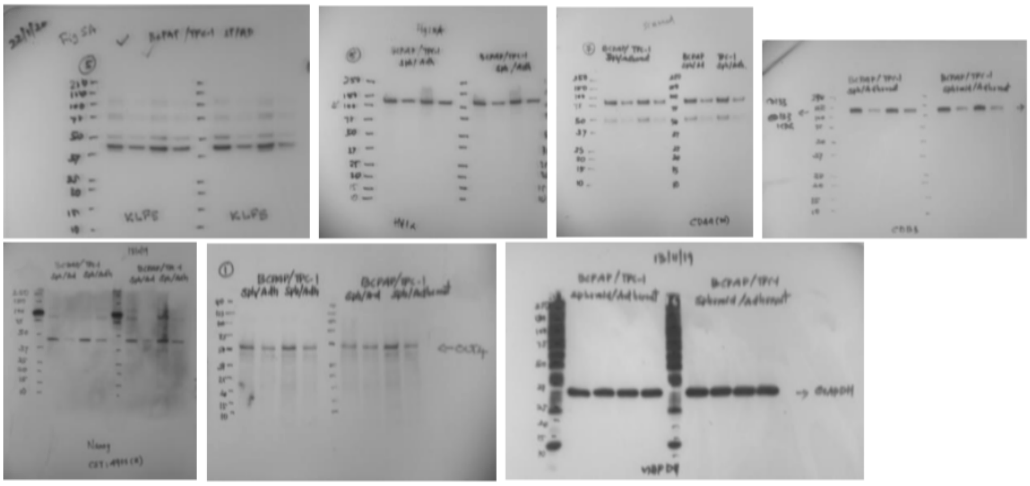

Figure 5E

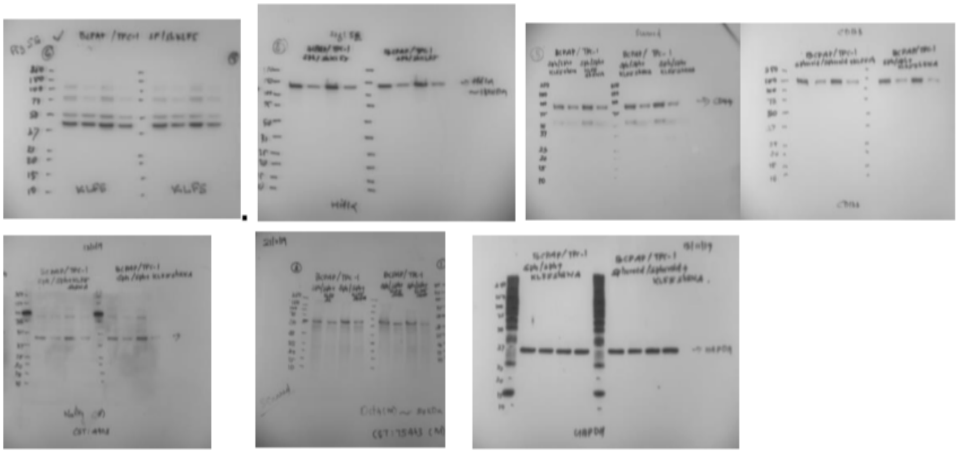

Figure 6D.

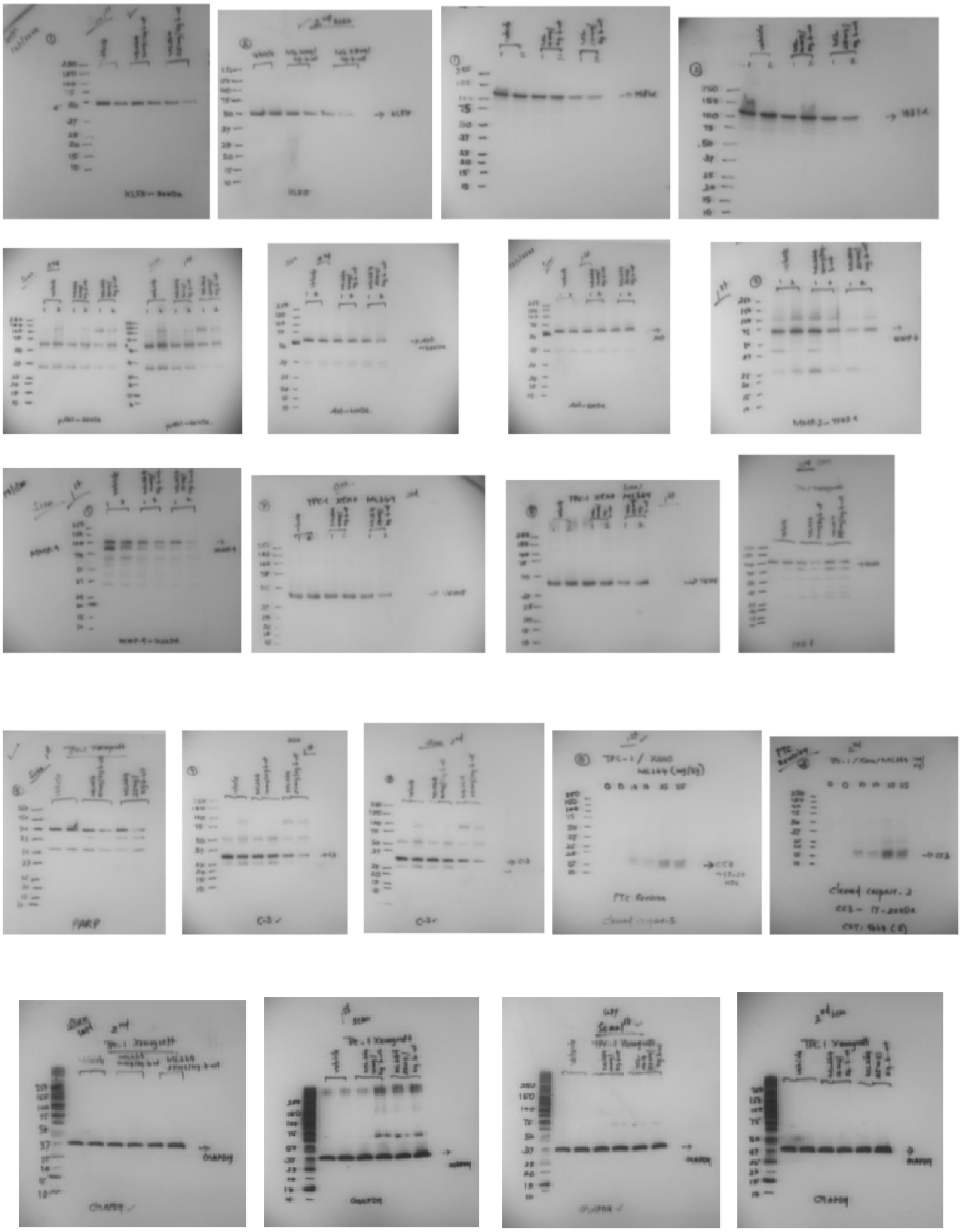

Supplement: Supplementary file 1 [file cancers-13-00185-s001.pdf]
